# Identification of Significant Features by the Global Mean Rank Test

**DOI:** 10.1371/journal.pone.0104504

**Published:** 2014-08-13

**Authors:** Martin Klammer, J. Nikolaj Dybowski, Daniel Hoffmann, Christoph Schaab

**Affiliations:** 1 Dept. of Bioinformatics, Evotec (München) GmbH, Martinsried, Germany; 2 Center for Medical Biotechnology, University of Duisburg-Essen, Essen, Germany; 3 Dept. Proteomics and Signal Transduction, Max-Plack Institute for Biochemistry, Martinsried, Germany; University of Westminster, United Kingdom

## Abstract

With the introduction of omics-technologies such as transcriptomics and proteomics, numerous methods for the reliable identification of significantly regulated features (genes, proteins, etc.) have been developed. Experimental practice requires these tests to successfully deal with conditions such as small numbers of replicates, missing values, non-normally distributed expression levels, and non-identical distributions of features. With the MeanRank test we aimed at developing a test that performs robustly under these conditions, while favorably scaling with the number of replicates. The test proposed here is a global one-sample location test, which is based on the mean ranks across replicates, and internally estimates and controls the false discovery rate. Furthermore, missing data is accounted for without the need of imputation. In extensive simulations comparing MeanRank to other frequently used methods, we found that it performs well with small and large numbers of replicates, feature dependent variance between replicates, and variable regulation across features on simulation data and a recent two-color microarray spike-in dataset. The tests were then used to identify significant changes in the phosphoproteomes of cancer cells induced by the kinase inhibitors *erlotinib* and 3-MB-PP1 in two independently published mass spectrometry-based studies. MeanRank outperformed the other global rank-based methods applied in this study. Compared to the popular Significance Analysis of Microarrays and Linear Models for Microarray methods, MeanRank performed similar or better. Furthermore, MeanRank exhibits more consistent behavior regarding the degree of regulation and is robust against the choice of preprocessing methods. MeanRank does not require any imputation of missing values, is easy to understand, and yields results that are easy to interpret. The software implementing the algorithm is freely available for academic and commercial use.

## Introduction

Today, omics-technologies are capable of generating vast amounts of data. Typical microarray experiments measure the abundance of thousands of features. With recent advances in the field of mass spectrometry (MS), over 10,000 proteins can currently be measured in cell systems [1], while recent studies identified even more phosphorylation sites through quantitative phosphoproteomics [2]–[4].

Many of these microarray and proteomics studies include the detection of differentially regulated features as core step in the data analysis. For data with thousands of features, the false discovery rate (FDR), defined as the expected number of false positive features among those reported as significant, has to be controlled [5]. However, strong control of the FDR reduces the rate of true positive features (TPR) discovered. The problem is often aggravated by experimental designs with small numbers of replicates. Further complications arise from missing data, especially common in MS-based shot-gun proteomics experiments. Microarray technologies often produce non-normally distributed expression levels and non-identical distributions between genes [6].

In principle, single-feature hypothesis tests like Student's *t*-test or the Wilcoxon rank-sum test can be applied to assess the significance of each feature, if results are corrected for multiple testing, e.g. by Benjamini-Hochberg (BH) [7] or the family-wise error rate (FWER) [8] procedures. However, when applied to data with only few replicates, these approaches are lacking statistical power, due to difficulties in estimating variance. Tusher *et al.* developed the Significance Analysis of Microarrays (SAM) [9], a more sophisticated method based on a modification of the *t*-statistic. The FDR is controlled by a permutation-based approach and adjusted using an estimate of the fraction of truly unregulated features. Moreover, SAM employs k-nearest-neighbor (k-NN) imputation to replace missing data. A similar approach is taken by empirical Bayes methods. Linear Models of Microarrays (LIMMA), for example, uses a moderated *t*-statistics, in which the estimated sample variance is shrunk towards a pooled estimate across all features [6].

Recently, methods applying a global approach, rather than determining significance on a feature-by-feature basis, were proposed. These methods take into account the entire dataset at once and thus avoid the difficult task of estimating the variance of each feature. Zhou *et al.* proposed a rank-based, global one-sample location test, which performs very well for small numbers of replicates and internally controls the FDR [10]. However, this global rank test requires features to consistently rank high or low across all replicates. The RankProducts test [11] is based on a similar global approach, but the ranks of each feature are multiplied. The FDR is then estimated numerically using random rank matrices.

The MeanRank test presented here borrows concepts of the GlobalRank and RankProducts tests, but uses a different test statistics and a different method for estimating the null-distribution. In the following, we describe the concept of MeanRank, including its handling of missing data. While we focus on the one-sample case in the main text, extensions to the two-sample case are discussed in File S1. The one-sample location test problem is equivalent to the paired-difference test problem for dependent samples. Paired samples are very common in proteomics experiments, which often apply labeling methods such as SILAC or iTRAQ, but also in transcriptomics (e.g. two-color microarray). We then present an extensive simulation study, in which the performance of MeanRank is compared to the previously mentioned tests, the t-test, and the Wilcoxon signed-rank test. In order to demonstrate the value of MeanRank, it is compared to SAM and LIMMA on the ‘Ag-Spike’ two-color microarray spike-in data set recently published by Zhu *et al.* [12]. Finally, MeanRank and SAM are applied to datasets of two published phosphoproteomics-studies.

## Results and Discussion

### Simulated data

In order to evaluate the performance of the MeanRank test and to compared it with various other tests, we performed an extensive simulation study extending the range of scenarios found in comparable publications [10], [13], [14] by including more parameters and wider ranges of replicates and methods. The advantage of simulations is that underlying statistical properties are known and, thus, the performance of different hypothesis tests can be compared under various conditions. In the first set of simulations we assessed the performance of the one-sample location tests for different sampling distribution parameters. Simulation parameters were strength of regulation (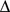), within-feature variance (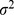) – both of which were either held constant or chosen to be variable – and the presence of missing values. These parameters were combined to generate different simulation scenarios. We calculated the performance for an increasing number of replicates for the respective scenarios. The parameters were deliberately chosen to simulate experiments with hard-to-identify regulated features to investigate the added power over a wide range of additional replicates. With the chosen settings, a true positive rate (TPR) of 1.0 should not be achieved easily.

**Figure 1:**
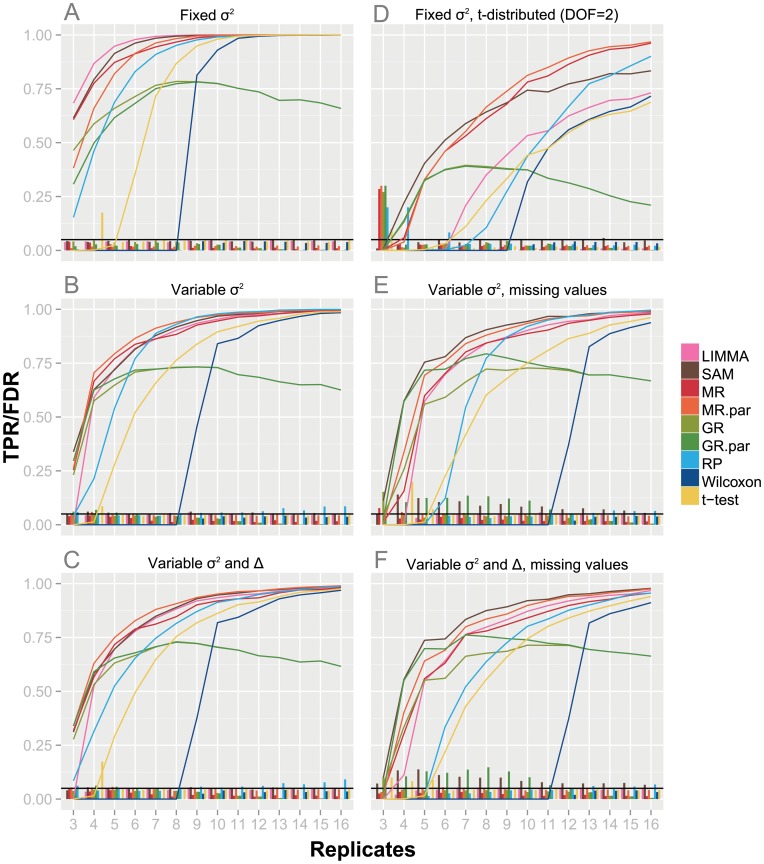
Performance on simulated data. Performance plot of one-sample significance tests under different simulation settings. Traces show the true positive rate (TPR) of the respective tests for a given number of replicates. Bars at bottom denote the false discovery rate (FDR). TPR and FDR are averaged over ten independent simulations. All tests were set to control the FDR at 0.05.

The simplest simulation setting assumes a constant variance and strength of regulation. Figure 1A shows TPR and FDR achieved by the tests when 3,600 unregulated features were sampled with constant 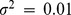 and 400 regulated features were sampled with a constant shift 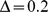. The leading method in this setting is LIMMA, followed closely by SAM, and then the non-parametric MeanRank (MR). This top-group clearly outperforms the other methods. The parametric MeanRank test (MR.par) has a somewhat lower power for data with less than five replicates in this specific simulation setting. The power of the GlobalRank tests (GR and GR.par) does not scale with the number of replicates, but reaches its maximum performance at nine replicates. Additional replicates will even lead to a loss in power. This behavior is expected, because with a growing number of replicates it becomes less likely for a regulated feature to consistently rank top or bottom. Similar to the parametric MeanRank (MR.par), the parametric GlobalRank (GR.par) is less powerful than its non-parametric counterpart for less than five replicates. In contrast to the GlobalRank, the power of the RankProducts (RP) scales well with the number of replicates, but it is less powerful for experiments with small number of replicates. The TPR curves of GlobalRank and RankProducts underline the initial motivation of developing the MeanRank test, i.e. combining the strengths of both tests without inheriting their shortcomings. The *t*-test shows significant lower TPR, most likely due to variance estimation issues, especially evident at very small number of replicates. As an example of a non-parametric, rank-based test that does not belong to the class of global approaches, we included the Wilcoxon signed-rank test. Because of the discreteness of the test statistics, it is not surprising that a minimum of nine replicates is required to identify any significantly regulated feature after multiple hypothesis testing correction. For eleven or more replicates the TPR approaches the TPR of the other tests beside the GlobalRank tests. All tests correctly control the FDR at the pre-specified level of 0.05.

Next, we investigated the scenario with feature dependent variable variance, which is frequently observed in omics data due to the dependence of the variance on the signal intensity [15]. Overall the tests display a similar behavior as in simulations with constant variance (Fig. 1B). However, while the overall TPR is slightly lower for most tests with variable 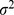, the parametric MeanRank and GlobalRank tests seem to be largely unaffected. Thus, the discrepancy between the parametric and non-parametric versions, which was observed for small number of replicates, disappears. Furthermore, MeanRank has a slightly higher overall TPR than SAM or LIMMA under these simulation conditions. The small gain in power for the *t*-test results from features with small variance caused by the variable 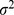 setting.

We then combined the variable variance 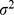 with a variable regulation strength 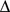, reflecting the complex response of systems to perturbations, e.g. of cells to drug treatment. There is a further loss in power across all tests, since some of the regulated features are hidden in the background noise (Fig. 1C). The parametric MeanRank performs best across all replicate numbers. The non-parametric MeanRank, SAM and LIMMA, exhibit comparable but slightly reduced power. In general, the behavior of all tests is similar to the previous simulation (Fig. 1B).

When using heavy-tailed distributions, such as a *t*-distribution, SAM and MeanRank exhibit similar power until up to seven replicates. However, while MeanRank progresses to a TPR of 1.0 for 15 replicates, SAM has by then just reached TPR 0.8 and almost levels off. (Fig. 1D). The power of LIMMA is considerably reduced compared to the previous scenarios and is comparable to the power of the *t*-test. The GlobalRank shows particular problems with this setting, achieving a TPR of merely 0.4, before starting to drop. The RankProducts even falls behind the *t*-test for less than nine replicates.

Missing data are common in technologies such as MS-based shotgun proteomics, thus in the next set of simulations we introduced missing values combined with variable variance 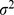 (Fig. 1E). It should be noted, that SAM is the only method used that does not handle missing data intrinsically. Instead, it employs a k-NN imputation prior to the actual significance analysis. In terms of power, parametric and non-parametric MeanRank together with SAM and LIMMA delivered the best results. For small numbers of replicates, the power of GlobalRank was comparable to that of MeanRank and SAM. However, SAM and the parametric GlobalRank systematically underestimated the FDR.

We additionally simulated the effect of missing values on data with both variable variance 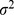 and shift 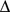 (Figure 1F). Here, the parametric and non-parametric MeanRank, SAM and LIMMA perform best with respect to the TPR. As in the previous scenario, SAM always underestimated the FDR considerably. In order to investigate whether the violations of FDR threshold observed for SAM were due to imputation, we also applied the other tests to the imputed data (see Figure S1). This resulted in similar behavior: a general violation of the FDR threshold, accompanied by a slightly higher TPR. Although it can be argued, that this is not a problem of SAM *per se*, the inability of handling missing data makes imputation inevitable.

Zhou *et al.* [10] stated that, in contrast to single-feature analysis methods, large numbers of features are advantageous for global methods and will lead to increased statistical power. We tested whether this applies to MeanRank, by altering the proportion of regulated and background features for a constant number of six replicates (see Figure S2). The hypothesis was confirmed, revealing that the rank-based tests (MeanRank, GlobalRank, and RankProducts) possess more power when the proportion of regulated to background features is small. The opposite is true for the single-feature-based tests, such as *t*-test, SAM and LIMMA. Despite experiencing a loss of power over an increasing fraction of regulated features, MeanRank always met the desired FDR threshold, while GlobalRank increasingly violated this threshold.

The simulations show that the parametric MeanRank generally had a higher power than the non-parametric version. Thus, we only used the parametric test in the following real data experiments. In the following, we applied parametric MeanRank, SAM and LIMMA, i.e. the tests showing the best performance in the above simulations, to microarray spike-in data and finally to real experimental datasets, for which, of course, the identity of truly regulated features is not known. However, since we showed that all tests with exception of SAM meet the pre-specified FDR in a series of different simulation scenarios, we can judge the performance of the test by evaluating the number of regulated features identified.

### Microarray spike-in data

**Figure 2:**
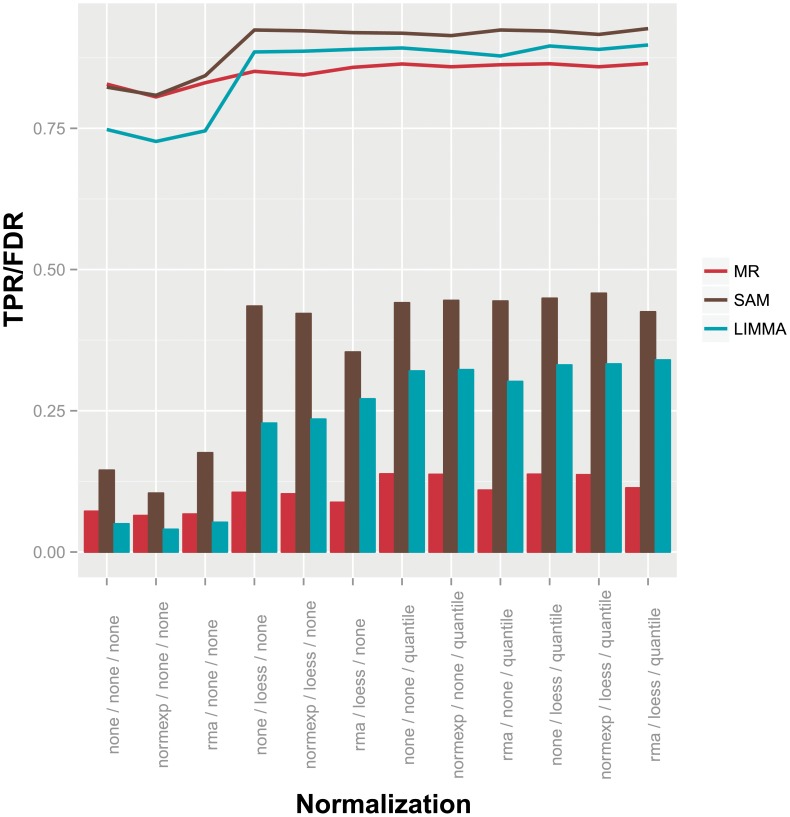
Performance on spike-in data. Performance comparison of MeanRank (red), SAM (brown), and LIMMA (cyan) on the ‘Ag-Spike’ microarray dataset [12]. TPR and FDR shown by lines and bars, respectively. Different combinations of preprocessing investigated by the authors of the original study are shown on the x-axis.

**Figure 3:**
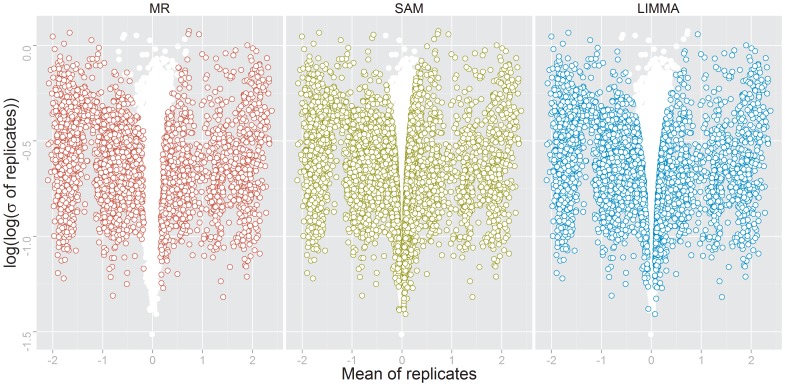
Volcano plot of spike-in data. Volcano plot of the ‘Ag-Spike’ data, background corrected by *normexp* and normalized with *loess*. This combination of preprocessing steps was found to deliver the best performance by the authors of the original study [12]. Genes are represented as points. Non-differentially expressed genes are scattered around Mean = 0 on the x-axis. Differentially expressed genes, as identified by the respective methods are colored.

Spike-in datasets are well-suited for the comparison of significance analysis methods, since the identity of truly regulated features is known before-hand. Here, we used the Agilent two-color microarray spike-in dataset (‘Ag-Spike’) consisting of 1300 differentially expressed and 2500 background cRNAs across 12 replicates [12]. In their study the authors explored different combinations of preprocessing methods (background correction, within-, between-array normalization) in order to identify optimal preprocessing routes for the detection of differentially expressed genes using LIMMA. We used the published preprocessed data to compare performance of the parametric MeanRank test with that of SAM and LIMMA. Figure 2 shows the true positive and false discovery rates of the three methods on the differently preprocessed spike-in data. Most notably, the rank-based approach of MeanRank is very robust against changes in preprocessing: *CV*_TPR_ = 0.04% and *CV*_FDR_ = 0.75% compared to SAM (*CV*_TPR_ = 0.21%, *CV*_FDR_ = 5.02%) or LIMMA (*CV*_TPR_ = 0.54% and *CV*_FDR_ = 6.02%). Slight variation is still introduced by methods applying local corrections, thus causing rank alterations (e.g. normalization *loess*). MeanRank on average identifies 2691 positives, 2354 (87%) of which are identified in all twelve preprocessing scenarios. The number of positives identified by SAM (4119) and LIMMA (3246) are higher on average, but clearly more dependent on the preprocessing protocol, with the number of constantly identified features being 2413 (59%) and 1989 (61%), respectively. This behavior is in line with the observations of Zhu *et al.*, who in a prior study found that the preprocessing protocol has a great impact on the performance of methods for detection of differentially expressed features [16]. The power of MeanRank is comparable to that of SAM and LIMMA, when none or only minimal efforts of normalization are made. Additional preprocessing steps result in greater power for SAM and LIMMA, however at the cost of an under estimated FDR. Zhu *et al.* found that a combination of background correction by *normexp* and within-array normalization using *loess* yields the best result. This measure looks at the true positive and corresponding false positive rates given the absolute value of the test statistic. Hence, the correct estimation of the FDR is not taken into account. Figure 3 shows volcano plots of the *normexp*-corrected and *loess*-normalized spike-in data and highlights differentially expressed features as identified by the different tests. The column-like structure of data points on the x-axis reflects the levels of spike-in (see Figure S3). The largest column centered at zero contains features not regulated. SAM and LIMMA, in contrast to the MeanRank test, tend to produce more false positives as the feature variance decreases.

### Phosphoproteomics data of *erlotinib*-treated AML cells

**Figure 4:**
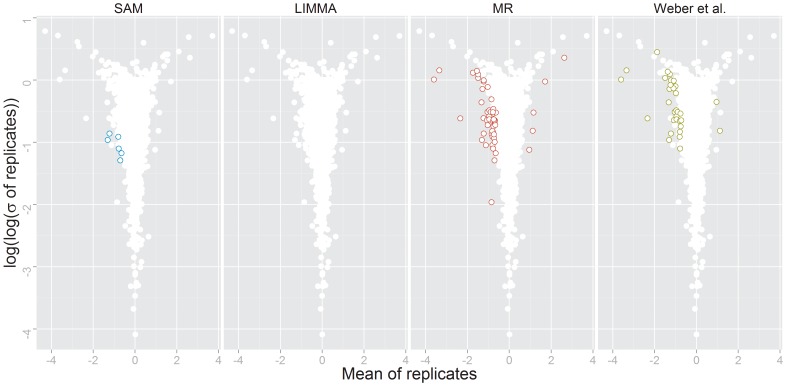
Volcano plot of AML data. Volcano plot of the phosphoproteomic data published by Weber *et al.* [17]. Significantly regulated phosphorylation sites are shown by colored circles as identified by SAM (left), the MeanRank test (right center), and in the original study (right).

We applied MeanRank, SAM and LIMMA to phosphoproteomics data published by Weber *et al.* [17]. The authors of that study performed SILAC-based, large-scale, quantitative mass spectrometry analyses of KG1 acute myeloid leukemia cells treated with the small molecule tyrosine-kinase inhibitor *erlotinib*, which mainly targets the *epidermal growth factor receptor* (EGFR). In their subsequent significance analysis of ratios of *erlotinib versus* control treatment the authors applied the RankProducts test to identify 33 significantly (FDR 0.05) regulated class-I sites (i.e. phosphorylation sites identified with high confidence). Prior to testing, ratios of class-I sites were log_10_-transformed and subjected to sample-wise median normalization (cf. [18]). The MeanRank test yielded 57 significantly regulated phosphorylation sites at FDR 0.05, including 24 of the 33 sites published by Weber *et al*. (Fig. 4, File S2). Of the remaining 9 sites, 8 had a local FDR smaller than 0.07, thus missing the significance criterion only marginally. 27 of the additional 33 sites identified by MeanRank had a missing ratio, emphasizing the tolerance of the test towards incomplete data. SAM identified only 5 sites as significantly regulated, while LIMMA did not identify any significantly regulated phosphorylation sites at all. The sites newly identified by MeanRank are located on 29 different proteins. Most of these proteins are annotated as being involved in the *cell surface receptor signaling pathway* (GO:0007166). Weber *et al.* further found that most site-specific repression of phosphoserines by *erlotinib* occurred on proteins involved in mRNA translation control. Supporting this finding, the MeanRank test also identified several transcription factors (GTF2B, GTF2F1, GTF3C1, DEAF1, and TCF12) to be significantly regulated upon treatment. In addition, we identified 6 additional phosphotyrosines sites. As the primary targets of erlotinib are tyrosine kinases, this significant relative enrichment (Fisher's exact test *p*<9.5⋅10^−6^) compared to the proportion of phosphotyrosins in the full dataset supports the findings of MeanRank. One of the sites that has not been identified as significantly regulated in the original paper is Tyr427 on the SHC-transforming protein 1 (Shc1). Tyr427 is phosphorylated *in-vitro* by Src kinase and *in-vivo* in EGF-stimulated cells [19]. Phosphorylated Shc1 forms a complex with Grb2 which in turn activates Ras signaling [20]. By down-regulation of Tyr427 on Shc1, erlotinib treatment inhibits the transmission of growth signals to the Ras signaling cascade.

### Phosphoproteomics data upon reactivation of Plk1

We investigated the behavior of MeanRank and SAM on data from a second phosphoproteomics study. Here, telomerase-expressing human retinal pigment epithelial (hTERT-RPE) cells expressing an analog-sensitive Plk1 mutant (Plk1^as^) were treated with the bulky kinase inhibitor 3-MB-PP1 [21]. 3-MB-PP1 inhibits the mutant kinase Plk1^as^ harboring an enlarged catalytic pocket, but not wild-type Plk1. This allowed the investigation of downstream effects upon Plk1 reactivation by inhibitor wash-out. The dataset contained four biological replicates with a total of around 20,000 identified phosphorylation sites. In this analysis, we considered only sites with values present in all four replicates in order to avoid having to impute data for SAM analysis. This left around 5,200 phosphosites to be tested for significant regulation upon inhibitor wash-out. Since SAM requires proper pre-processing, the data were log_10_-transformed and median normalized (cf. [18]).

**Figure 5:**
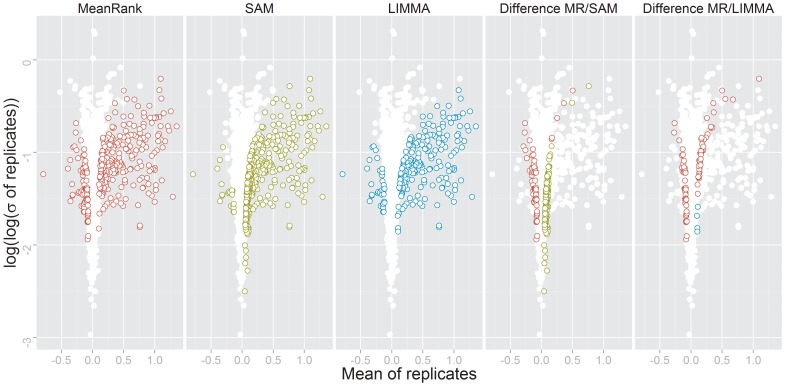
Volcano plot of Plk1-kinase-inhibited cells data. Volcano plot of the phosphoproteomic data of cells treated with an Plk1 tyrosine kinase inhibitor *versus* control [21]. Significantly regulated phosphorylation sites shown in colored circles as identified by MeanRank test, SAM, LIMMA (from left). The two rightmost volcano plots shows differences in detected phosphorylation sites by MeanRank/SAM and MeanRank/LIMMA.

While MeanRank identified 313 significantly regulated phosphorylation sites (FDR 0.05), SAM reported a slightly higher number of 359 significant sites for the same FDR level (File S3). The overlap of the reported significant features was 249. SAM identified more significantly up-regulated features than MeanRank, most of which exhibit low variance and low mean regulation (Fig. 5). SAM found 152 sites that were less than 1.5-fold up-regulated on a linear scale; MeanRank only 45. In contrast, SAM found only 8 sites that were less than 1.5-fold down-regulated (linear scale), while MeanRank reported 61. MeanRank draws a more consistent threshold between significantly up-, and down-regulated features than SAM.

LIMMA reports 229 significantly regulated phosphorylation sites, 225 are also identified by MeanRank. Similar to MeanRank, LIMMA mainly reports sites with mean regulation stronger than ±1.5-fold on a linear scale. Only 23 sites with down-regulation of less than 1.5-fold and 18 with an up-regulation of larger than 1.5-fold are reported.

Generally, it appears that SAM puts more emphasis on variance and MeanRank more emphasis on the level of regulation. This is reflected in the shape of the region within the volcanoplot, in which the significant features are located (Fig. 5): While the significantly regulated features identified by MeanRank can be separated from the background features almost by a straight line, those identified by SAM exhibit a rather curved threshold line. LIMMA behaved similar to MeanRank, however identifying slightly less significantly regulated sites. Since the simulation study suggests that both tests comply with the pre-specified FDR level when applied to four-replicate experiments, it can be argued that at least 95% of the phosphosites reported as being significantly regulated by either test are in fact true positives. While all three tests perform well and have a high overlap, either might be more suitable depending on the application.

### Two-sample test

Established methods such as SAM and LIMMA support two-sample comparison experiments. The MeanRank test can be extended to accommodate two-sample comparisons by basically transforming the two-sample into a one-sample problem. To do so, we create a difference matrix by calculating the difference of each possible pair from both groups. Here we assume that the data is log-transformed. The calculations of the mean ranks is then performed on the difference matrix in the same way as for the one-sample test. Since the columns of the difference matrix are not independent anymore, the dependency structure has to be taken into account when estimating the null distribution. We found that although the test generally performs well in terms of power compared to SAM for most cases in our simulations and spike-in microarray data [16] while reliably controlling the FDR, it is very conservative when applied to data with missing values. This can be explained by the way the difference matrix approach exaggerates the relative amount of missing values. The method and simulation setup is described in detail File S1 (see also Fig. S4 in File S1).

## Conclusions

The simulations showed that borrowing traits from both the GlobalRank and RankProducts methods strongly improved the power over either of the two tests in all simulated scenarios, while reliably estimating the FDR. All three tests are rank-based and use a global approach rather than testing feature-by-feature. The main differences of the MeanRank test compared to the other two tests are the test statistics and the methods for estimating the distribution under the null-hypothesis. We showed that this improves the power of the test with respect to the RankProducts test for low number of replicates and avoids a drop in power with increasing number of replicates in the case of the GlobalRank test.

While single-feature-based non-parametric tests, such as the Wilcoxon rank-sum or signed-rank tests, require nine or more replicates in order to identify any significant regulated feature at all, this is not the case for global rank-based tests. The fixed 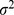 simulations showed, that the non-parametric MeanRank test identifies more than 60% of the true positives for three replicates.

The parametric and non-parametric MeanRank tests performed comparably to SAM and LIMMA in most simulation scenarios. While SAM and LIMMA performed slightly better in the case of fixed 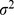 simulations, MeanRank had a slightly higher power in the cases with variable 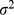 and both variable 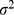 and 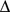.

When introducing missing data, our simulations suggest that SAM tends to underestimate the FDR, since missing values have to be imputed. This naturally raises concerns when applying SAM – and thus imputation – to data resulting from technologies like MS-based shotgun proteomics, regularly producing missing values. The matter is further complicated by the fact that different imputation methods (k-nearest-neighbor, singular value decomposition, multiple imputation, etc.) can deliver deviating results [22]. These aspects have to be considered, when applying SAM to data with missing values, while the MeanRank test offers a convenient way to entirely avoid imputation. However if, under certain conditions, imputation delivered results close to the ground truth, the power of any test would increase. A distinct advantage of the MeanRank test lies in the decoupling of significance testing and imputation procedures, leaving the freedom of choice with the researcher. If the data were not normally distributed but followed a heavy-tailed distribution such as the *t*-distribution with few degrees of freedom, the one-sample MeanRank test showed a better performance than SAM and in particular LIMMA, especially for experiments with many replicates.

The global nature of the MeanRank test leads to a loss in power when a very large fraction of features is truly regulated. However, several studies suggest that the fraction of differentially regulated features is often lower than 10% [17], [21]. In fact, given such experiments, our simulations show that the MeanRank test has an advantage over single-feature-based tests like SAM and LIMMA. A notable practical advantage of MeanRank over other methods such as SAM is that normalization of samples is not necessary due to its rank-based nature. This is advantageous because normalization can have a direct influence on the results, as was demonstrated by our comparison based in the ‘Ag-Spike’ data. Here, MeanRank, in contrast to SAM or LIMMA, produced very stable results, independent of the preprocessing steps applied. SAM attempts to determine the proportion 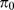 of true null hypotheses in the dataset in order to adjust the false discovery rate [5]. This usually leads to more positive calls; however, the estimation of 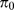 is not robust against small variations in the data and depends strongly on the preprocessing applied. Since the FDR estimation of the MeanRank test is rather conservative, an implementation of a similar estimation could help to further improve the test with respect to statistical power. However, we deliberately omitted 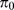 estimation because of the described inconsistent behavior also seen in other studies [23].

In summary, the key advantages of the MeanRank test compared to other tests are: a comparable or even superior power in detecting regulated features without underestimation of the FDR, the possibility to analyze data with missing values without the necessity for imputation; the robustness with respect to preprocessing. Although we focused on the one-sample test in the main text, a two-sample version of the test is also available and described in File S1. One-sample location tests are particular important for the analysis of proteomics data which often uses labeling methods such as SILAC or iTRAQ, but also for the analysis of two-color microarrays. Furthermore, they can be applied to paired two-sample test problems emerging, for example, if matched tumor and normal tissues are measured across many patients. The MeanRank test is not limited to testing the significance of gene- or protein regulation. As no strong assumptions about the underlying distributions are made for the non-parametric test, inference about statistically significant differences between groups could, in principle, be made for any kind of ordinal features. Furthermore, MeanRank is freely available and can be used by anyone without any restrictions, whereas SAM is patented and requires proper licensing. For most experiments, running the MeanRank test is a matter of seconds, and can be performed on standard computers (see Table S1).

Finally, we would like to emphasize the intuitiveness of our test. MeanRank is easy to understand, easy to implement, does not require any parameter optimization and yields results that are easy to interpret.

## Materials and Methods

### MeanRank test

Given a matrix 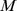 of 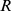 columns (replicates) and 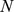 rows (features, e.g. genes, proteins, phosphorylation sites). Let 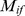 be the value of feature *f* (with 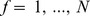) in replicate i (with 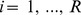). Based on this matrix *M*, for each replicate *i* the ranks 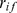 of each feature *f* within this replicate and across all features can be determined by sorting the values in each replicate. This is in contrast to the Wilcoxon signed-rank test, for which the ranks are calculated across the replicates. Then the mean rank is calculated for each feature across all replicates. Similar to the approach of Zhou *et al.*, [10], the mean rank statistic is motivated by the random ordering theorem, i.e. under the null hypothesis *H*_0_ that no feature is either up- or down-regulated, it is very unlikely that a feature ranks consistently high or low across all replicates. Therefore no extreme (very large or very small) mean rank values can be expected. In contrast to Zhou *et al.*, who require features to rank top or bottom consistently across all replicates, the mean rank statistic may tolerate some moderate outliers.

For simplicity, we will focus on the detection of significantly down-regulated features in the following, but the same approach is applicable for up-regulated features by simply switching the signs of all values. The mean rank test proceeds in these steps:

1. Sort features ascendingly by their values within each replicate

2. Calculate mean rank as
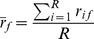
(1)


3. Sort values 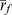 ascendingly (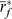) and identify the top *n* as significantly down-regulated

In case of tied ranks, the values are left in the original order, receiving ascending ranks. The list of significantly regulated features depends on the value of *n*, which has to be chosen to meet the specified FDR. The FDR is defined as the expected fraction of false positives among the reported positives. Following the approach of Zhou *et al.* [10], we denote 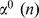 the expected number of false positives among the top *n* features. The FDR is thus
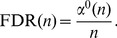
(2)


As the true form of the null distribution is not known, we have to estimate a null distribution either parametrically or non-parametrically. For a parametric estimate, we assume that the mean ranks of the null distribution follow a Bates distribution, i.e. the distribution of the mean of statistically independent uniformly distributed random variables. The cumulative distribution function is defined as:
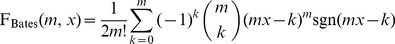
(3)where *m* is the number of random variables and *x* is the mean of the random variables scaled to the interval (0, 1), and 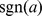 is −1 for 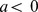, 0 for 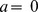, and 1 for 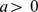. The expected number of false positives is then calculated as:
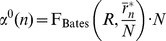
(4)


Non-parametric estimation of 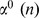 follows Zhou *et al.*, assuming a non-regulated feature has the same probability of ranking top or bottom [10]. Thus, the null distribution is independent of whether the features are sorted in ascending or descending order, or – analogously – whether the features values have a positive or negative sign. Consequently, 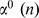 can be estimated by alternately flipping the signs of the ratios of the replicates, calculating the flipped mean ranks 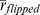 on this flipped data, and counting the number of values in 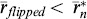 (see pseudo-code in File S1).

### Missing data

To account for missing data values, which are especially common in MS-based proteomics experiments, the equation in step (2) of the algorithm has to be modified to
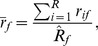
(5)where 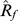 is the number of present data values of the respective feature and 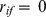 if the value is missing. It has to be ensured that missing values are not considered in the ranking process and consequently do not receive a rank (they are ignored completely). The FDR estimation has to be modified as well, as there are now features with different numbers of data values in the dataset. Thus, the parametric estimation of false positives has to be modified to
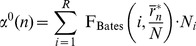
(6)where *N_i_* is the number of features with *i* data values present. The non-parametric estimation of 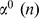 remains largely unchanged, however, one has to ensure that features unaffected by sign-flipping are excluded. This occurs when sign-flipping is by chance applied only to values that are missing. The resulting feature would be unchanged and receive the same ranks and subsequently mean rank, after flipping.

### Simulations

Artificial data was generated by sampling from various distributions. The background distribution (unregulated data) containing 3600 features was drawn from a normal distribution with zero mean (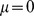) and standard deviation 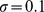; 400 regulated features (80 up- and 320 down-regulated) were sampled from normal distributions with shifted means (shift 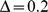). We investigated the performance with an increasing number of replicates (3 to 15). The described settings were then altered to simulate variable variance by drawing 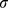 from a uniform distribution 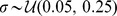) in combination with constant regulation strength between features (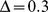) and variable regulation 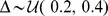. Missing data were introduced by randomly discarding 20% of data points while ensuring that at least two thirds of the data points were present for each feature. In simulations of non-normal data we sampled features from a *t*-distribution with two degrees of freedom. Whenever imputation of missing values was applied, the k-nearest neighbor (*k* = 10) method was used.

Significance analyses by RankProducts, SAM and LIMMA were performed using the *RankProd* [24], *samr*, and *limma* packages of Bionconductor [25] for R [26], respectively. The global rank method by Zhou *et al*. [10] was implemented by the authors. *t*-test and Wilcoxon signed-rank test *p*-values were BH corrected for multiple hypothesis testing [7].

## Supporting Information

Figure S1 **Performance on simulated data using imputation.** Performance plot of tests for one-sample simulation data with missing data imputed by k-nearest-neighbor (k-NN) with *k* = 10.
(TIF)

Figure S2 **Performance for different fractions of regulated and unregulated features.** Performance with fixed number of replicates (*R* = 6), over a varying fraction of regulated features to background features.
(TIF)

Figure S3 **Volcano plot highlighting spike-in concentrations.** Volcano plot of the ‘Ag-Spike’ data, colored by fold-change of spike-in.
(TIF)

Table S1 **Computational performance of the MeanRank test.** Computation time and memory usage shown in seconds and megabytes, respectively. Measurements were performed on a single core of an Intel i5 2400, with 3.1 GHz.
(PDF)

File S1 **Additional information on the MeanRank test.** Pseudo-code for the one-sample variant, and methods and simulation results for the two-sample variant of the MeanRank test.
(PDF)

File S2 **Significance testing results of AML data published by Weber** ***et al*** **.** Data originally published by Weber *et al.* [17] with additional significance testing performed using the parametric MeanRank test, SAM and LIMMA.
(TXT)

File S3 **Significance testing results of Plk1 data published by Oppermann** ***et al*** **.** Data originally published by Oppermann *et al.* [21] with additional significance testing performed using the parametric MeanRank test, SAM and LIMMA.
(TXT)
