# Supplementary figures and images for: Identification of Significant Features by the Global Mean Rank Test
Source: PLoS One. 2014 Aug 13;9(8):e104504. doi: 10.1371/journal.pone.0104504 (PMC4132091; doi:10.1371/journal.pone.0104504)

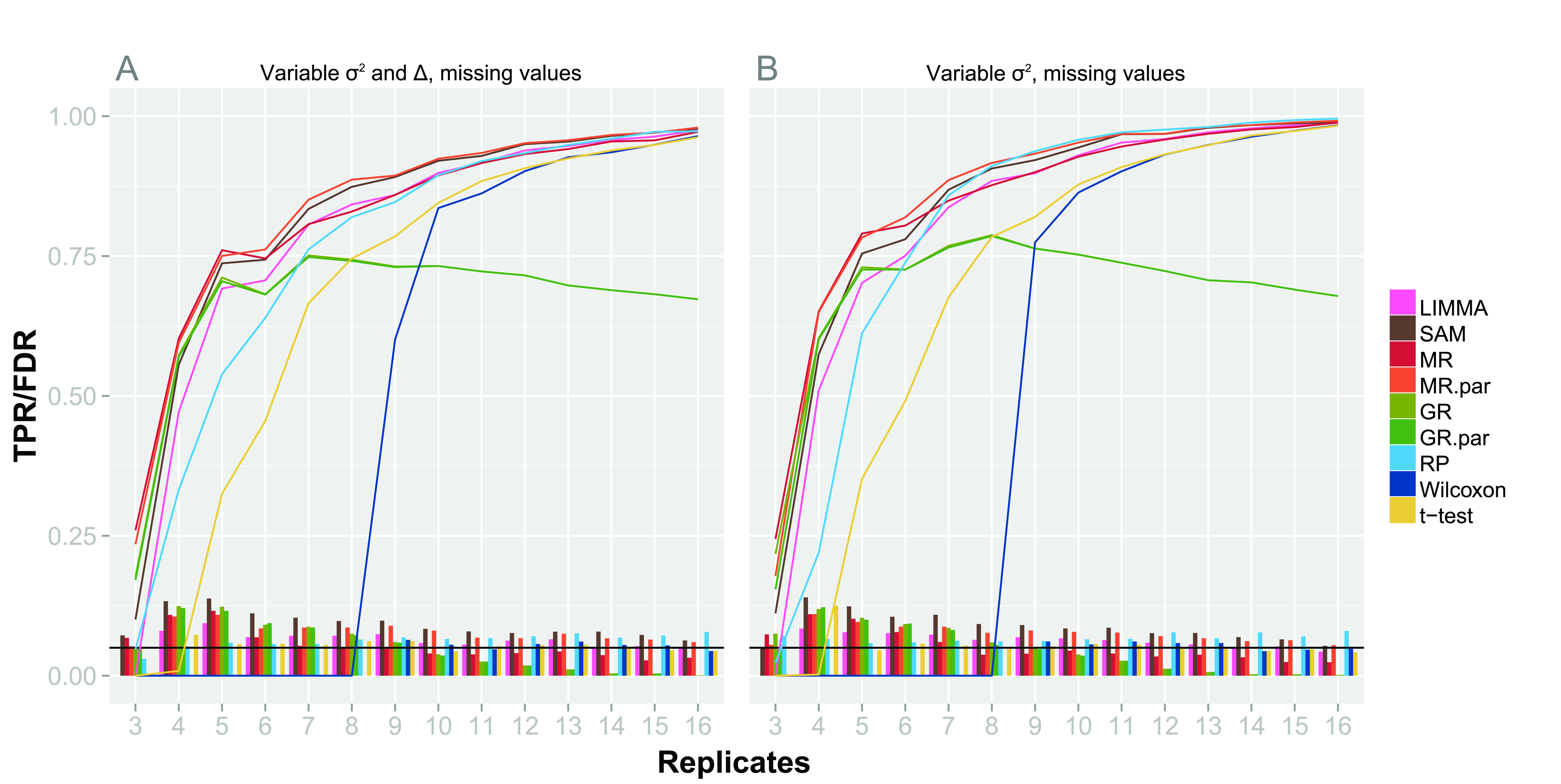

Supplement: Figure S1 — Performance on simulated data using imputation. Performance plot of tests for one-sample simulation data with missing data imputed by k-nearest-neighbor (k-NN) with k = 10. (TIF) [file pone.0104504.s001.tif]

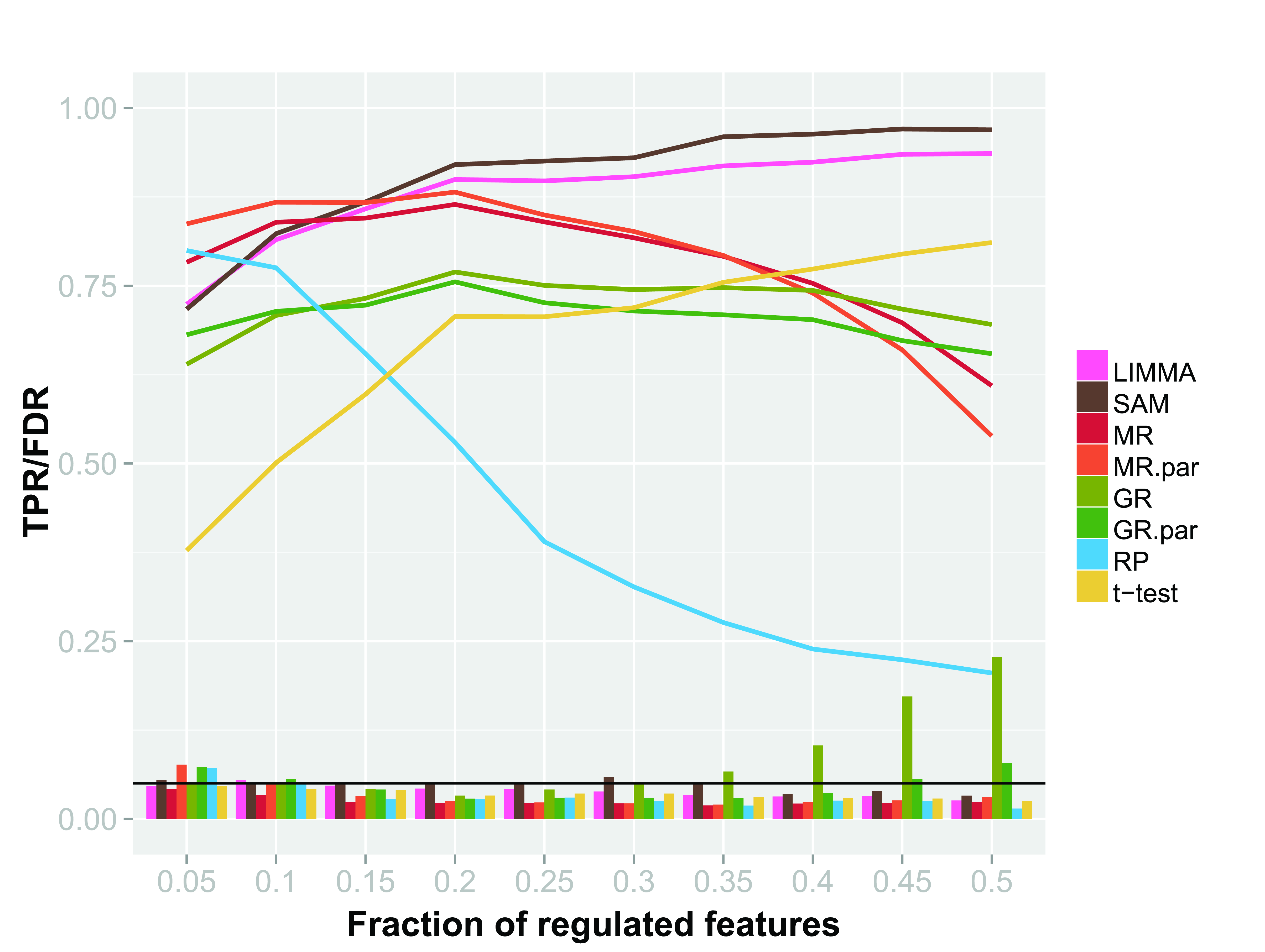

Supplement: Figure S2 — Performance for different fractions of regulated and unregulated features. Performance with fixed number of replicates (R = 6), over a varying fraction of regulated features to background features. (TIF) [file pone.0104504.s002.tif]

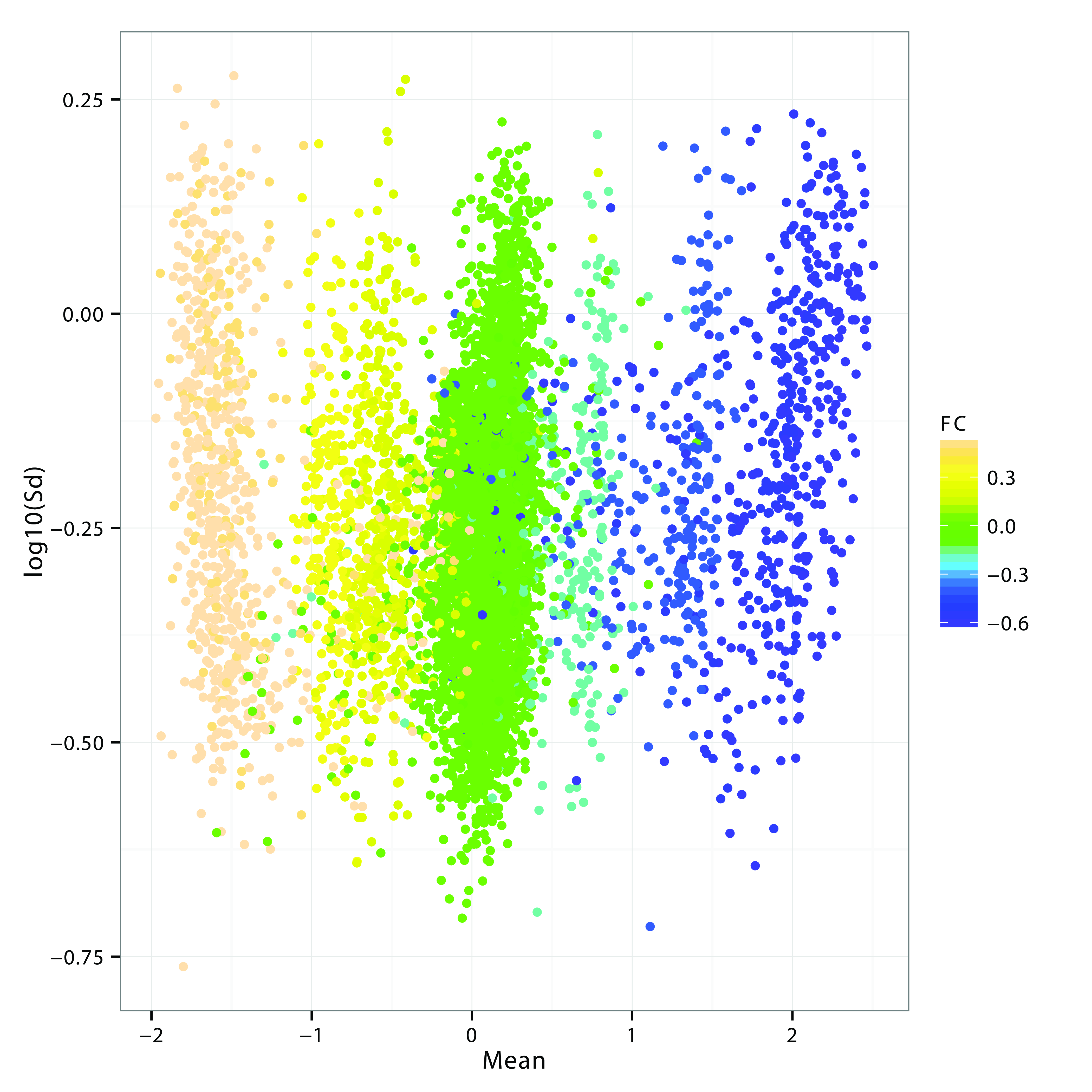

Supplement: Figure S3 — Volcano plot highlighting spike-in concentrations. Volcano plot of the ‘Ag-Spike’ data, colored by fold-change of spike-in. (TIF) [file pone.0104504.s003.tif]
