# Supplementary material for: Identification of Significant Features by the Global Mean Rank Test
Source: PLoS One. 2014 Aug 13;9(8):e104504. doi: 10.1371/journal.pone.0104504 (PMC4132091; doi:10.1371/journal.pone.0104504)
Supplement: Table S1 — Computational performance of the MeanRank test. Computation time and memory usage shown in seconds and megabytes, respectively. Measurements were performed on a single core of an Intel i5 2400, with 3.1 GHz. (PDF) [file pone.0104504.s004.pdf]

	R replicates			
	R=5		R=30	
N features	Parametric	Non-parametric	Parametric	Non-parametric
$N = 1.000$	$<$ 1s / $<$ 1MB	$<$ 1s / $<$ 1MB	3s / $<$ 1MB	2s / $<$ 1MB
$N = 10.000$	8s / $<$ 1MB	20s / 1MB	45s / 2MB	184s / 18MB
