# Supplementary material for: Identification of Significant Features by the Global Mean Rank Test
Source: PLoS One. 2014 Aug 13;9(8):e104504. doi: 10.1371/journal.pone.0104504 (PMC4132091; doi:10.1371/journal.pone.0104504)
Supplement: File S1 — Additional information on the MeanRank test. Pseudo-code for the one-sample variant, and methods and simulation results for the two-sample variant of the MeanRank test. (PDF) [file pone.0104504.s005.pdf]

File S1: Identification of Significant Features by the Global Mean Rank Test

Pseudocode of one-sample MeanRank algorithm

Algorithm S1 Calculation of α^0, the expected number of false discoveries.

```
M = matrix holding features and replicates
r̄_sorted = vector of sorted mean ranks
α⁰_sum = vector of length N
for all s ∈ Cartesian product {−1, +1}^R without{(−1, . . . , −1), (+1, . . . , +1)} do
    M_flipped = multiply values in columns (i = 1, . . . , R) of M with values of s_i
    r̄ = mean ranks ofM_flipped
    for i = 1, . . . , N do
        α⁰_sum(i)+ = count r̄_flipped < r̄_i,sorted
    end for
end for
α⁰ = α⁰_sum / number of flips
```

Two-sample Mean Rank test

The two-sample version of the proposed test is similar to the one-sample case, with only a few modifications. For the two-sample case, the two input matrices, M_1 and M_2, hold the data from two different groups, e.g. treated and untreated. Both must have the same number of features N, but the number of replicates may be different (R_1 and R_2, respectively). As the aim of two-sample tests is to find differentially regulated features between two groups, we create a difference matrix prior to step 1. in Equation (1) in the main article. This difference matrix contains all possible $R_1 \cdot R_2$ pair-wise differences between the two data matrices. Note, that often values should be log-transformed to achieve a symmetric distribution of differences. The ranks are then calculated on the difference matrix and steps 2. and 3. of Equation (1) are performed using this $R_1 \cdot R_2$ matrix of difference ranks.

The Bates distribution cannot be used for the parametric estimation of α^0, as the $R_1 \cdot R_2$ columns of the difference rank matrix are not independent. Thus, the null distribution has to be determined numerically. This is done by generating two random data matrices (sampled from a standard normal distribution) with R_1 and R_2 columns, respectively, and very large N ($\geq 100,000$). The difference rank matrix is then calculated as described above, and the empirical distribution of the resulting mean rank values is determined, which can then be used instead of $\mathrm{F}_{\mathrm{Bates}}$ function to estimate α^0.

The non-parametric estimation of α^0 has to be modified for the two-sample test, as well. Instead of performing sign flipping to estimate false positives, we now randomize the group association and calculate α^0 accordingly.

Additional simulations were carried out to assess the performance of the two-sample MeanRank, and compare it to the two-sample versions of SAM, RankProducts and the t-test. A two-sample version of GlobalRank does not exist. In line with the results of the previous one-sample simulations, MeanRank and SAM performed better than the other methods (see Figure S4 in this document). In simulations with normally distributed data and no missing values MeanRank and SAM showed comparable power and met the FDR level. In the case of simulation data sampled from a non-normal (Student's-t) distribution, the TPR of the parametric MeanRank test drops as the parametric FDR estimation involves the assumption of normal distributions. The introduction of missing values led to a drop in power for MeanRank. This can be explained by the way the difference-rank matrix is calculated. Since each subtraction involving

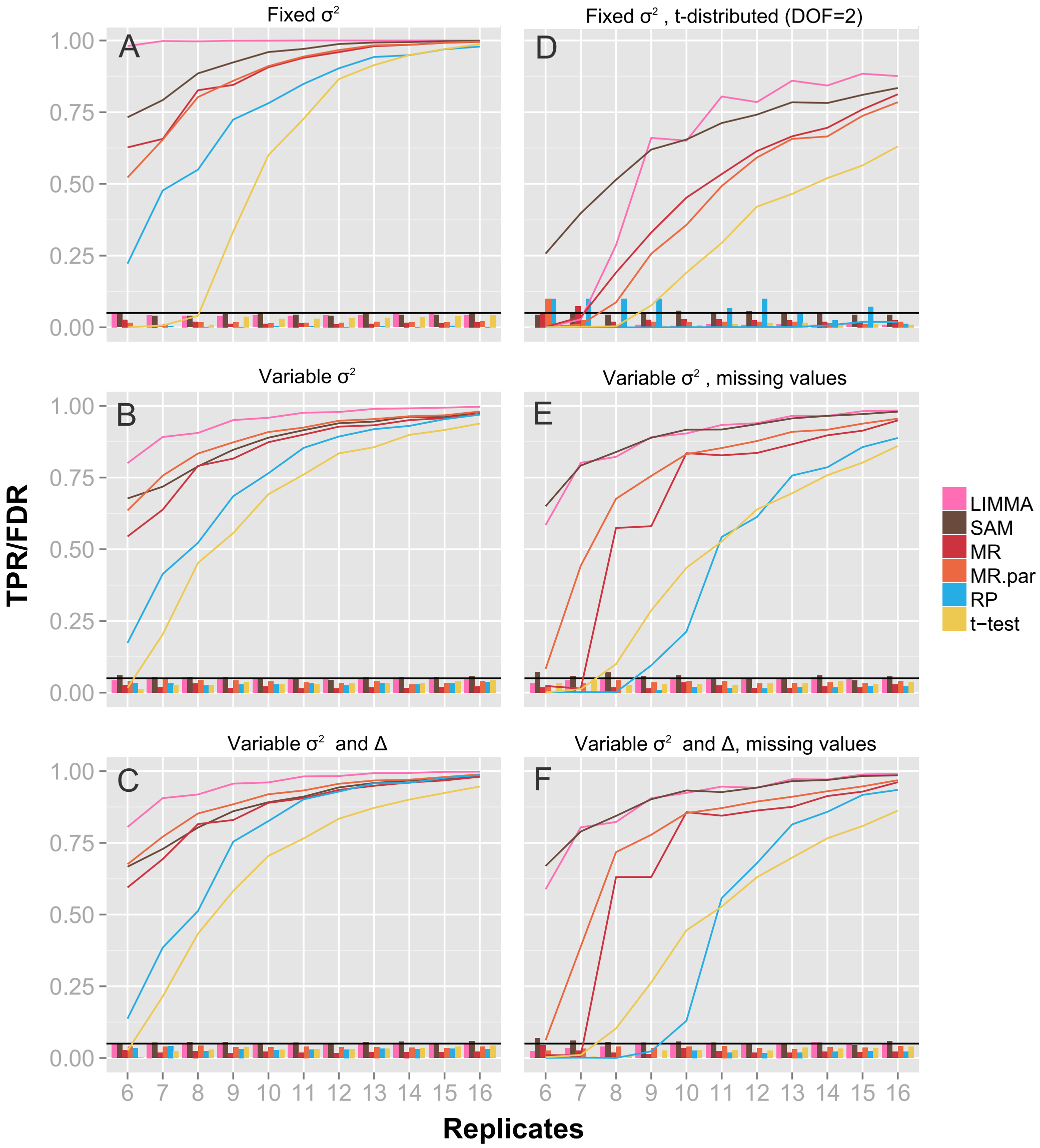

Figure S4. Performance of two-sample tests on simulated data. Performance plot of two-sample significance tests under different simulation settings. Traces show the true positive rate (TPR) of the respective tests for a given number of replicates. Bars denote the false discovery rate (FDR). TPR and FDR are averaged over ten independent simulations. All tests were set to control the FDR at 0.05.

a missing value again produces a missing value, the proportion of missings in the difference-rank matrix is larger than in the initial data matrices M_1 and M_2. However, when using the same imputed data as SAM, both methods achieve a similar TPR, while facing the drawbacks of missing value imputation (e.g. underestimation of FDR).
